# Predictors of tuberculosis infection among adults visiting anti-retroviral treatment center at east and west Gojjam, northwest, Ethiopia, 2017

**DOI:** 10.1186/s12879-020-05290-2

**Published:** 2020-08-12

**Authors:** Habtamu Belew, Moges Wubie, Getaye Tizazu, Abebaw Bitew, Tesfa Birlew

**Affiliations:** 1 Department of Medical Laboratory Science, College of Medicine and Health Sciences, Debre Markos University, 269, Debre Markos, Ethiopia; 2 Department of Public Health, College of Medicine and Health Sciences, Debre Markos University, 269, Debre Markos, Ethiopia

**Keywords:** Predictors, Tuberculosis, HIV infection, Anti-retroviral treatment

## Abstract

**Background:**

Tuberculosis is a serious health risk, for people living with human immune deficiency virus worldwide, and the burden of TB/HIV infection is still high in Ethiopia in particular. Therefore, the aim of this study was to determine the predictors of tuberculosis infection among adults visiting anti-retroviral treatment center in East and West Gojjam, northwest, Ethiopia.

**Methods:**

Institution based unmatched case-control study was employed to determine the predictors of tuberculosis infection among adults visiting anti-retroviral treatment center in east and west Gojjam, Northwest, Ethiopia from March 7–April 15, 2017. Just about 552 participants were participated in the study (139 Cases and 413 controls). Cases were confirmed with active TB and infected with HIV, and controls were HIV positive adults with non-TB. All cases in each health facility who confirmed by acid-fast bacilli, culture and gene expert were considered as TB positive. However, controls were selected by using simple random sampling technique through the above diagnostic criteria and the data were collected with Face to face interview as well as patient medical record were utilized, and the quality of the data were assured, checked, coded, cleaned and entered in EPI-Data version 3.1 and exported to SPSS version 20 for the analysis.

**Result:**

Of the total sample (556), just about 552(99.2%) were participated in the study. 47.5% were females and 58.9% were rural dweller. Behavioral and modifiable biological risk factors: alcohol users (AOR = 2.33; 95%CI:1.34,4.07), BMI < 18.5 kg/^m2^ (AOR = 3.03;95%CI:1.79,5.14), CD4 count ≤200 cells/μl (AOR = 2.34;95%CI:1.89,2.79) and between 201 and 499 cells/μl (AOR = 2.63; 95%CI: 1.01,6.84), bedridden and ambulatory (AOR = 3.3;95%CI:1.70,6.29 and AOR = 8.2;95%CI:4.34,15.64), respectively. TB history in the family (AOR = 3.00; 95%CI: 1.57, 5.74) were predictors for TB infection. Taking CPT (AOR = 0.36; 95%CI: 0.21, 0.62) and having early WHO clinical stage I or II (AOR = 0.34; 95%CI: 0.20, 0.56) had protective effect against TB infection.

**Conclusion:**

From this study, it has been concluded that alcohol users, BMI < 18.5 kg/m^2^, CD4 count < 499 cells/μl, bedridden and ambulatory and TB history were predictors for TB-HIV co-infected adults. Strengthen screening more frequently, CPT Prophlaxysis and treated promptly important to reduce TB co-morbidity.

## Background

Tuberculosis (TB) is often the commonest infection to develop among people living with human immunodeficiency virus (PLWHIV). Among the world’s 22 high-burden tuberculosis countries, Ethiopia ranks the seventh in worldwide and third in Africa [1]. HIV pandemic presents a massive challenge to the control of TB at all levels. In high HIV prevalence population, TB is a principal cause of morbidity and death, and HIV is driving the tuberculosis epidemic in many countries, especially in sub-Saharan Africa [2]. TB is leading cause of death among PLWHIV, which known as “fatal duo” as HIV reduces the body’s defense system and makes them more horizontal to TB infection. On the other hand, TB increases the development of HIV to acquired immune deficiency syndrome (AIDS) stages [3]. The association between HIV and TB often described as a co-epidemic; they are far harsher together than either disease alone in developing countries many people infected with HIV catch TB as the first sign of AIDS [4]. TB is the most common opportunistic infection in PLWHIV worldwide [5]. It is the first presenting sign in the majority of AIDS patients, including those who are taking antiretroviral treatment (ART). Despite major reductions with ART, however, risk of TB remains high in Africa [6, 7]. Globally, an estimated 13% of TB cases co-infected with HIV, and the bacteria accelerate the progression of HIV to AIDS [7]. Without proper treatment, 90% of PLWHIV die within months of contracting TB. The current challenge is to find ways of preventing both TB and HIV, and to improve diagnosis and management of co-infection, so the HIV/AIDS and *Mycobacterium tuberculosis* co-infection is one of the major global challenges in the prevention of TB and accounts for 33% HIV related deaths [6, 8]. Progress against HIV has far outstripped global efforts to tackle TB. In 2014, for the first time in decades, TB killed more people than any other infectious disease in the world. HIV infection is the strongest known risk factor for TB and estimated that there were 1.4 million TB deaths in 2015, and an additional 0.4 million co-morbid deaths [8–10]. HIV-infected people have an annual risk of 5–15% of developing active TB once infected, and have a much higher risk of developing active TB than HIV-negative individuals [10–13]. In resource-limited settings, the healthcare systems flurry under by the preventive, therapeutic and diagnostic challenges of the HIV–TB syndemic. However, HIV is not the only determinant for developing TB, various other factors: socio-demographic, clinical, life style and environmental are determinant for TB in HIV positives [14, 15]. In Ethiopia, the ministry of health attempt to improve community level access to care by decentralizing TB/HIV service from hospital to health centers and build up referral systems as well as step up standard operating procedures and standards of care to improve the quality [16, 17], but the burden of TB/HIV infection is still high. This unexpected scale of the outbreak of HIV related tuberculosis demands intensive and urgent action, but also there is no such study in the study area conducted before. Therefore, the aim of this study is to identify predictors of tuberculosis infection among HIV infected adults’ visiting anti-retroviral treatment center in public health institutions in East and West Gojjam Zone, Northwest Ethiopia, 2017.

## Methods

### Study area and period

The study conducted in the public health institutions in East Gojjam (Debre Markos Referral Hospital, Debre Markos health center, Dejene health center, Bichena health center, Lummame health center & Yebokela health center). From West Gojjam, Finotselam hospital was included. The study area was selected according to available number of TB/HIV co-infection in the area and computerized documentation system in the health facilities. The study was conducted from March 7 to April 15, 2017 in Northwest, Ethiopia.

#### Study design

Institutional based unmatched case-control study design was conducted.

#### Source population

All adult HIV infected adults recorded on ART from public health institutions.

#### Sampling population

The cases were both active TB and HIV infected adults with TB treatment during the data collection period and registered on TB clinic. Controls were only HIV infected adults and negative for TB and registered on ART.

### Eligibility criteria

#### Inclusion criteria

All adult living with HIV and TB/HIV co-infected individuals with pulmonary tuberculosis, extra Pulmonary tuberculosis and being ≥15 years old and having record on ART and TB clinic in public health institutions in east and west Gojjam were included in the study.

#### Exclusion criteria

Those patients, who were seriously ill, had a mental problem and with suspected but unconfirmed TB exclude from this study.

### Sample size determination

As the investigation was unmatched case-control study, the sample size would require for using two-population proportion formula to achieve statistically significance results. Therefore, a sample was calculated by taking into account the major exposure variables, and using epi-info version 7. Among the exposure variables CD_4_ count, isoniazed preventive therapy (IPT) and cotrimoxazole preventive therapy (CPT) were evaluated. The variables that were given the largest sample size in this case was CD_4_ count selected as the main exposure variables which given the most favorable sample size, and the study were planned to have 80% statistical power with a level of significance at 5% (two-sided) for a case to control ratio of 1:3. Assuming the proportion of low CD_4_ cell count is 5.6% for the controls and 13.9% for the cases [14] and allowing 5% of non- response rate the resulted sample size were 576.

### Sampling procedure

Regarding to cases a base line data was obtained from the governmental hospitals and public health centers. Two hospitals and five health centers in east and west Gojjam were found to be eligible by simple random sampling.

The principal investigator extracts the required data from the ART and TB registries for the identification of cases and controls. All TB–HIV co-infected adults attending HIV care clinics that could diagnose by direct microscopy, culture, Gene expert and fine needle aspiration and confirmed for TB positive. TB-HIV co-infected adults, who would receive TB treatment and fulfill inclusion criteria at the time of data collection, were included as a case in the study. However, controls were adequate to be sampled and confirmed tuberculosis negative according to the guideline diagnosed; lists of controls were prepared using unique identification numbers from records found in ART clinics and selected by simple random sampling technique by using computer-generated random numbers. Medical registration number used to select controls that fulfill inclusion criteria after giving unique identification numbers in increasing order.

### Study variables

#### Dependent variable

Status of tuberculosis among HIV infected adults.

#### Independent variables

A Socio demographic characteristic includes (sex, age, marital status, residence, level of education, type of occupation and monthly income). Environmental factors (separate kitchen, Family size, number of windows in the house, the types of floor in the house), Host or behavioral related factors (chewing khat, cigarette smoking, drinking alcohol, history of TB in the past, history of asthma, history of TB in the family, & history of diabetic mellitus). Clinical variables (WHO clinical stage, hemoglobin level, IPT prophylaxis, CPT prophylaxis, level of CD_4_ count, functional status of the patient and level of BMI) were included.

### Operational definitions

**Substance abuse** (chewing khat, alcohol consumption and smoking) defined as an individual who is currently using the substance or has a history of regular substance abuse.

**Active tuberculosis** is *Mycobacterium tuberculosis* disease which is in active state in any part of the body as determined by either, a smear microscopy, culture or molecular taken from any source in the person’s body tests positive for tuberculosis and the person has not completed the appropriate prescribed course of medication for active tuberculosis during the study.

**Smear positive pulmonary tuberculosis (PTB**^+^**)** diagnosed if single sputum smear examination positive for Acid Fast Bacilli (AFB) by direct microscopy, culture, Gene expert and laboratory confirmation of HIV infection. **Extra-pulmonary tuberculosis** diagnosed if one specimen from an extra-pulmonary site culture-positive for *Mycobacterium tuberculosis* or smear positive for AFB or histological or strong clinical evidence consistent with active extra-pulmonary tuberculosis and laboratory confirmation of HIV infection or strong clinical evidence of HIV infection and decision by a clinician to treat with a full course of anti -tuberculosis chemotherapy [14].

### Data collection instruments

To determine the predictors of tuberculosis among HIV infected adults, semi-structured questionnaires were used to collect data in study participants from selected public health institutions in East and West Gojjam Zone from primary and secondary sources. The data were collected by face-to-face interview of patients and extracted from ART card and logbooks. Five trained clinical diploma nurses who were not in charge of an HIV and TB care clinic conducted the interview with study participants using questionnaires.

### Data quality control

Before data collection period, data collectors trained about the objective of the research. The principal investigators were given the training about the objective of the study and data collection system by using semi-structured questionnaires in a one-day period. The questionnaires would prepare in English, translated into Amharic and back translated into English to check consistency. Pre-test carried out on 16 PLWHIV positive adults in Amanual health center in Machakele woreda to familiarize the interviewer with the instrument and to check the coherence. To keep the quality of data, principal investigator was checking the questionnaires for its completeness in each day.

### Data processing and analysis

The data were checked for completeness and consistencies, and cleaned, coded and entered using Epi-Data V.3.1computer program and exported to statistical package for social science (SPSS) version 20 for analysis. The whole data were cleaned to minimize data entry errors and observed the unities of the data. Inconsistencies were verified using cross tabulation. Descriptive frequency statistics were run to see the general distribution of the data. Frequencies and proportions used to describe the study subjects in relation to the study variables. Multiple logistic regressions were used to review the predictors of TB in HIV infected adults as the most important statistical method of analysis by using backward logistic regression variable selection technique*.* All explanatory variables that were associated with the outcome variable in bivariate analysis with *p*-value of < 0.25 [36] were candidate in the initial logistic regression models of multivariate analysis. The crude and adjusted odds ratios together with their corresponding 95% confidence intervals were computed. A *P*-value < 0.05 considered as statistically significant with the outcome variable.

## Results

Among 556 selected study participants, 552 study subjects (139 cases (25.2%) and 413 controls (72%) were responded and the overall response rate were 99.2% (96.5% for cases and 99% for controls). A higher percentage of women observed in the study participants 301(54.5%). Sixty-six (47.5%) of the cases and 235 (57%) of the controls were females. The median age of the participants was 32.5 years and IQR of 25–41.75. Seventy-six (54.7%) of the cases and 249 (60.3%) of the controls were rural residents. More than 2/3 of the study participants were completed primary school and above; 66.9% in the cases and 71.67% in the controls (Table 1).

**Table 1:** Descriptive statistics of socio demographic characteristics for factors of TB infection among HIV positive adults at east and west Gojjam, Northwest, Ethiopia, 2017

Variables	Cases n (%) ***n*** = 139	Controls n (%) ***n*** = 413
Sex		
Male	73(52.5)	178(43)
Female	66(47.5)	235(57)
Age		
15–34 years	72(51.8)	224(54.2)
≥35 years	67(48.2)	189(45.8)
Residence		
Urban	63(45.3)	164(39.7)
Rural	76(54.7)	249(60.3)
Marital status		
Single	28(20.14)	68(16.5)
Married	52(37.41)	219(53)
Divorced	32(23.02)	75(18.2)
Widowed	27(19.43)	51(12.3)
Level of education		
No education	46(33.1)	117(28.33)
Primary	48(34.5)	112(27.12)
Secondary	26(18.7)	100(24.21)
Above 12	19(13.7)	84(20.34)
Occupation		
Employed	18(12.95)	56(13.6)
Unemployed	17(12.23)	52(12.6)
Others	104(74.82)	305(73.8)
Monthly income		
< 650Birr	62(44.6)	122(29.5)
≥650Birr	77(55.4)	291(70.5)

Others indicate merchant, farmer, and student

### Distribution of behavioral and environmental factors for TB among HIV infected adults

Thirty one (22.3%) of the cases and 48 (11.6%) of the controls were chewing chat. Among the environmental variables 87 (62.6%) of the cases and 300 (72.6%) of the controls were lived in mud/soil types of floor in the house (Table 2).

**Table 2:** Distribution of behavioral and environmental factors for TB infection among HIV-infected adults at east and west Gojjam, Northwest, Ethiopia, 2017

Variables	Cases n (%) ***N*** = 139	Controls n (%) ***N*** = 413
Khat chewing		
Yes	31(22.3)	48(11.6)
No	108(77.7)	365(88.4)
Smoking		
Yes	16(11.5)	31(7.5)
No	123(88.5)	382(92.5)
Drinking alcohol		
Yes	49(35.3)	71(17.2)
No	90(64.7)	342(82.8)
TB in the past		
Yes	17(12.2)	58(14)
No	122(87.8)	355(86)
Asthma		
Yes	24(17.3)	40(9.7)
No	115(82.7)	373(90.3)
Diabetic mellitus		
Yes	30(21.6)	60(14.5)
No	109(78.4)	353(85.5)
TB in the family		
Yes	33(23.7)	41(9.9)
No	106(76.3)	372(90.1)
Types of floor		
Mud/soil	87(62.6)	300(72.6)
Cement	52(37.4)	113(27.4)
Family size		
≤2	41(29.5)	106(25.7)
3–4	65(46.8)	201(48.7)
≥5	33(23.7)	106(25.6)
Separate kitchen		
Yes	91(65.5)	267(64.6)
No	48(34.5)	146(35.4)
No of window in the house		
0	24(17.3)	61(14.8)
1	57(41)	126(30.5)
≥2	58(41.7)	226(54.7)

### Clinical factors for TB infection among HIV positive adults

The median CD4 count of the study participants were 393.5 cells/μl and IQR of 210.5–624.00. Sixty-two (44.6%) of the cases and 103 (39.5%) of the controls had CD_4_ cell counts ≤200 cells/μl. One hundred three (74%) of the cases and 125 (30.3%) of the controls had BMI of less than 18.5 m^2^/kg (Table 3).

**Table 3:** Clinical factors for TB infection among HIV positive adults at east and west Gojjam, northwest, Ethiopia, 2017

Variables	Cases n (%) *N* = 139	Controls n (%) *N* = 413
CD4 count		
≤ 200cells/μl	62(44.6)	64(15.5)
201-49cells/μl	62(44.6)	163(39.5)
≥ 500 cells/μl	15(10.8)	186(45)
Hemoglobin		
< 10 mg/dl	70(50.4)	80(19.4)
10–11.99 mg/dl	40(28.8)	99(24)
≥12 mg/dl	29(20.8)	234(56.6)
BMI		
< 18.5 m2/kg	103(74)	125(30.3)
≥18.5 m2/kg	36(26)	288(69.7)
Functional Status		
Bedridden	71(51.1)	36(8.7)
Ambulatory	33(23.7)	92(22.3)
Working	35(25.2)	285(69)
CPT prophlaxysis		
Yes	82(60)	328(79.4)
No	57((40)	85(20.6)
IPT prophylaxis		
Yes	42(30.2)	148(35.8)
No	97(69.8)	265(64.2)
WHO Staging		
Stage I/II	38(27.3)	273(66.1)
Stage III/IV	101(72.7)	140(33.9)

### Bivariate analysis for factors associated with TB infection among HIV positive adults in east and west Gojjam zone

The odds of TB infection among HIV infected adults were 2.83 times higher in previously TB history in the family as compared to non-TB history. CD_4_ count ≤200cells/μl and 201-499cells/μl (crude OR (COR) 2.55;95%CI 1.615,4.016 and 12.01;95%CI 6.388,22.588) times more likely exposed for TB infection among HIV positive adults as compared to CD_4_ count of ≥500cells/μl (Table 4).

**Table 4:** Bivariate logistic regression for socio-behavioral, environmental and clinical factors associated with TB infection among HIV positive adults at east and west Gojjam, Northwest, Ethiopia 2017

Variables	Cases n(%) *n* = 139	Controls n (%) *n* = 413	COR(95%CI)	*P*-value
Sex				
Male	73(52.5)	178(43)	1.46(0.99,2.147)	0.054
Female	66(47.5)	235(57)	1	
Age in years				
15–34	72(51.8)	224(54.2)	.907(0.617,1.332)	0.618
≥35	67(48.2)	189(45.8)	1	
Residence				
Urban	63(45.3)	164(40)	1.25(0.854,1.855)	0.245
Rural	76(54.7)	249(60)	1	
Marital status				
Single	28(20.14)	68(16.5)	1.7(1.017,2.958)	0.043*
Married	52(37.41)	219(53)	0.96(0.527,1.766)	0.908
Divorced	32(23.02)	51(18.2)	0.78(0.410,1.477)	0.442
Widowed	27(19.43)	84(12.3)	1	
Level Education				
No education	46(33.1)	117(28.3)	0.92(0.567,1.483)	0.725
Primary	48((34.5)	112(27.1)	1.51(0.872,2.621)	0.141
Secondary	26(18.7)	100(24.2)	1.74(0.951,3.178)	0.073
Above 12	19(13.7)	84(20.4)	1	
Occupation				
Employed	18(12.95)	56(13.6)	0.98(0.459,2.108)	0.965
Unemployed	17(12.23)	52(12.6)	0.95(0.535,1.693)	0.867
Others	104(74.82)	305(73.8)	1	
Monthly income				
< 650	62(44.6)	122(29.5)	1.92(1.210,2.853	0.001*
≥650	77(55.4)	291(70.5)	1	
Chat chewing				
Yes	31(22.3)	48(11.6)	2.18(1.324,3.599)	0.002*
No	108(77.7)	365(88.4)	1	
Smoking				
Yes	16(11.5)	31(7.5)	1.6(0.848,3.030)	0.146
No	123(88.5)	382(92.5)	1	
Drinking alcohol				
Yes	49(35.3)	71(17.2)	2.6(1.703,4.039	0.0001*
No	90(64.7)	342(82.8)	1	
TB history past				
Yes	17(12.2)	58(14)	0.85(0.617,1.332)	0.618
No	122(87.8)	355(86)	1	
Asthma				
Yes	24(17.3)	40(9.7)	1.94(1.126,3.365)	0.017*
No	115(82.7)	373(90.3)	1	
Diabetic mellitus				
Yes	30(21.6)	60(14.5)	1.62(0.994,2.638)	0.053
No	109(78.4)	353(85.5)	1	
TB history in the family				
Yes	33(23.7)	41(9.9)	2.83(1.702,4.688)	0.0001*
No	106(76.3)	372(90.1)	1	
CD4 count				
≤ 200cells/μl	62(44.6)	64(15.5)	2.55(1.615,4.016)	0.0001*
201-49cells/μl	62(44.6)	163(39.5)	12(6.388,22.588)	0.0001*
≥ 500 cells/μl	15(10.8)	186(45)	1	
Hemoglobin				
< 10 mg/dl	70(50.4)	80(19.4)	2.17(1.33,3.526)	0.002*
10–11.99 mg/dl	40(28.8)	99(24)	7.06(4.275,11.66)	0.0001*
≥12 mg/dl	29(20.8)	234(56.6)	1	
BMI				
< 18.5 m2/kg	103(74)	125(30.3)	6.59(4.272,10.17)	0.0001*
≥18.5 m2/kg	36(26)	288(69.7)	1	
Functional Status				
Bedridden	71(51.1)	36(8.7)	5.5(3.126,9.672)	0.0001*
Ambulatory	33(23.7)	92(22.3)	16.1(9.425,27.365)	0.0001*
Working	35(25.2)	285(69)	1	
CPT prophlaxysis				
Yes	82(60)	328(79.4)	0.372(0.247,0.564)	0.0001*
No	57((40)	85(20.6)	1	
IPT prophlaxyis				
Yes	42(30.2)	148(35.8)	0.772(0.512,1.173)	0.228
No	97(69.8)	265(64.2)	1	
WHO Staging				
Stage IorII	38(27.3)	273(66.10)	0.193(0.126,0.295)	0.0001*
Stage IIIorIV	101(72.7)	140(33.9)	1	
Type of floor				
Mud/soil	87(62.6)	300(72.6)	0.63(0.420,0.946)	0.026*
Cement	52(37.4)	113(27.4)	1	
No of window In the house				
0	24(17.3)	61(14.8)	0.87(0.494,1.533)	0.629
1	57(41)	126(30.5)	1.53(0.881,2.666)	0.130
≥2	58(41.7)	226(54.7)	1	
Family size				
≤2	41(29.5)	106(25.7)	1.20(0.758,1.888)	0.442
3–4	65(46.8)	201(48.7)	1.24(0.730,2.114)	0.424
≥5	33(23.7)	106(25.6)	1	
Separate kitchen				
Yes	91(65.5)	267(64.6)	1.04(0.692,1.552)	0.863
No	48(34.5)	146(35.4)	1	

*COR* Crude odds ratio, *CI* Confidence interval, *CPT* Cotrimoxazole preventive therapy, *IPT* Isoniazid preventive therapy

*indicates that the variable entered into multiple logistic regressions or shows significant variation, value of n (%)

### Multivariate analysis for the predictors of TB infection among HIV infected adults in east and west Gojjam

Thirteen variables that indicated *P*-value ≤0.25 in the binary logistic regressions were marital status, monthly income, chewing chat, drinking alcohol, asthma, TB history in the family, CD_4_ cell count, Hgb level, BMI, functional status of the respondent, CPT prophlaxysis, WHO clinical stage and types of floor in the house were candidate for multivariate analysis. By using backward variable selection methods, a multivariate logistic regression model showed that seven variables were predictors for TB infection among HIV infected adults. From these factors, being patients who drink alcohol (AOR = 2.33; 95%CI: 1.34, 4.07) was independent predictor of increased TB infection as compared to patients that did not drink alcohol. However, WHO clinical stage I or II (AOR = 0.34; 95%CI: 0.20,0.56) and cotrimoxazole preventive therapy (AOR = 0.36; 95%CI: 0.21,0.62) had protective effect against tuberculosis infection. But, monthly income, chat chewing, hemoglobin level, asthma and types of floor in the house were lost their statistical significance in multivariate analysis (Table 5).

**Table 5:** Predictors of factors associated with TB infection among HIV positive adults visiting ant-retroviral treatment centers at east and west Gojjam, Northwest, Ethiopia, 2017

Variables	Cases *N* = 139	Controls *N* = 413	COR(95%CI)	AOR (95%CI)
Drinking alcohol				
Yes	49(35.3)	71(17.2)	2.62(1.70,4.04)	2.33(1.34,4.07)
No	90(64.7)	342(82.8)	1	1
TB history in the family				
Yes	33(23.7)	41(9.9)	2.83(1.70,4.69)	3.0(1.57,5.74)
No	106(76.3)	372(90.1)	1	1
WHO staging				
StageI/II	38(27.3)	273(66.1)	0.19(0.12,0.30)	0.34(0.20,0.56)
Stage III/IV	101(72.7)	140(33.9)	1	1
CPT prophlaxysis				
Yes	82(60)	328(79.4)	0.37(0.25,0.56)	0.36(0.21,0.62)
No	57(40)	85(20.6)	1	1
Functional status				
Bedridden	71(51.1)	36(8.7)	5.5(3.13,9.67) 16.1(9.43,27.4)	3.3(1.70,6.29)
Ambulatory	33(23.7)	92(22.3)	1	8.2(4.34,15.64)
Working	35(25.2)	285(69)		1
CD4 count				
≤200/μl	62(44.6)	64(15.5)	2.55(1.62,4.02)	2.34(1.89,2.79)
201-499 μl	62(44.6)	163(39.5)	12.01(6.39,22.6	2.63(1.01,6.84)
≥500 μl	15(10.8)	186(45)	1	1
BMI				
< 18.5 kg/^m2^	103(74)	125(30.3)	6.59(4.27,10.17	3.03(1.79,5.14)
≥18.5 kg/^m2^	36(26)	288(69.7)	1	1

*AOR* Adjusted OR, *COR* Crude odds ratio, *CI* Confidence interval, *CPT* Cotrimoxazole preventive therapy, *BMI* Body mass index

## Discussion

This study showed that HIV infected adults taking CPT and early WHO clinical stage I/ II were less likely to the incidence of TB infection. Although, drinking alcohol, TB history in the family, CD_4_ cell count of ≤200cells/μl and between 201 and 499 cells/μl, functional status of bedridden and ambulatory and having BMI of < 18.5 kg/^m2^ were predictors of expansion of TB infection among HIV infected adults.

Our findings show that alcohol drinkers were predictors of TB infection among HIV positive patients in this study. This was consistent with previous studies conducted in South India [18], West Africa [19] and Amhara Region [20]. Nevertheless, in contrast to the study conducted in Gambia [21] alcohol use is not associated with TB infection, this is because of socio-demography, sampling technique difference and sample size. Smoking was not associated with increased TB infection in this study; this is in agreement with previous study conducted in Addis Ababa [22]. However, in other study conducted in West Africa [19], which identified smoking as predictors for increased TB infection among HIV infected adults, this difference was probably due to sample size difference, social desirability bias where smokers deprived of their smoking status in this study. Previous TB history in the family found to be predictors for increased TB occurrence in the study; this is in line with the study conducted in Croatia, West Africa and southwest Ethiopia [19, 23, 24].

Low body mass index (LBMI< 18.5 kg/ m^2^) was associated with increased TB infection in this study; this is parallel to a study done in South India [18], Croatia [23], South Africa [25] and south central Ethiopia [26]. This might be due to the fact that under nutrition deteriorates the immunity level that increases the reactivation of latent tuberculosis infection. On the other hand, malnutrition can aggravate the immune deficiency and increase the risk of active TB. Building on the existing knowledge this study also found out that low body mass index increased the likelihood of tuberculosis infection [22, 27]. The CD_4_ count ≤200 cells/μl and between 201 and 499 cells/μl were about 2.34 and 2.63 times more likely to develop TB as compared to CD_4_ count ≥500 cells/μl respectively. This is comparable with previous reports conducted [14, 20, 24, 26, 28–33]. The most important predictors for the development of TB in HIV patients as it frequently reported in literatures are the immunological state of the person. Maintaining the CD4 positive cell level as high as possible in patients with advanced disease helps the person to have low risk of re-activation of tubercle bacilli [34]. This study confirm that patients with functional status bedridden and ambulatory were 3.3 and 8 times more likely predictors for the expansion of TB infection, this is related to other findings conducted in Hawassa, Addis Ababa, Northwest Ethiopia and Amhara Region [11, 20, 26, 32, 34]. This is because of the low immunity behavior of the participant during the study period.

In addition, our findings indicating that cotrimoxazole preventive therapy had protective effect against TB infection were also consistent with previous research reports [11, 14, 35, 36]. Cotrimoxazole preventive therapy has advantageous effect in increasing CD4 cell count as well falling viral load and CPT is reasonably priced and is generally well tolerated, this endorse TB/HIV associations should give priority high level coverage to the achievement of cotrimoxazole preventive therapy as they have proven usefulness in improving patients condition [27]. Different studies have shown that isonized preventive therapy reduces the risk of infection in people living with HIV however, differently in this study IPT has not associated with risk of TB, because of low coverage of IPT [31] and confounding factors in the study participants. WHO clinical staging and TB infection among HIV positive adults were significantly associated in the study. Those patients in WHO clinical stage I/ II were about 0.340 times less likely to develop TB as compared to those WHO clinical stages III/IV. This is consistent with the study conducted in Amhara Region [20]. Nevertheless, this result was not in line with study done in Tanzania [30] and south central Ethiopia [26]. The possible reason for the difference might be source population, sample size and socio demographic difference. This could make clear when the patients get early WHO stage that the immunity protective capacity will be highest which would make them protect to tuberculosis infection, as well as TB is one of the AIDS defining criteria to categorize the patients into the late WHO clinical staging which again was not addressed with this study. This study indicated determinants of TB infection among HIV positive adults visiting ant-retroviral treatment centers that would be significant for prioritizing TB screening, diagnosis, treatment and prevention. In addition, the study showed that cotrimoxazole preventive therapy and early WHO clinical stage I or II are protective effect for reducing TB infection among HIV positive adults in resources-limited settings [32, 33, 37, 38]. The study has the following limitations; case control study design could not set up temporal relationship and can only shows associations; it could not proof causations. Recall bias might have also affected the occurrence of information related to substance use such as cigarette smoking, chat chewing and alcohol consumption; also social desirability bias is unavoidable during data collection period.

## Conclusions

This study indicated that drinking alcohol, previous TB history in the family, functional status being bedridden and ambulatory, CD_4_ cell count <500cells/μl, BMI < 18.5 k g/^m2^ were predictors of increased tuberculosis infection among HIV infected adults visiting ant-retroviral treatment centers in East and West Gojjam Zone and attention is given for these predictors for TB diagnosis. However, this imitated that in early WHO clinical stag I or II and using cotrimoxazole preventive therapy is necessary to reduce the overall risk of TB infection among HIV positive individuals. Hence, these findings supply momentum to strengthen tracing of TB household and member contacts screening.

## Data Availability

The datasets used and/or analysed during the current study are available from the corresponding author on reasonable request. All relevant data are found in the manuscript.

## Abbreviations

AIDS: Acquired immunodeficiency syndrome
ART: Antiretroviral treatment
AOR: Adjusted odds ratio
BMI: Body mass index
CD_4_: Cluster of deffirnciation of t-lymphocyte cells
CPT: Cotrimoxazole preventive therapy
COR: Crude odds ratio
DMRH: Debre Marko’s Referral Hospital
DMU: Debre Marko’s University
EPHI: Ethiopian public health institute
FSDH: Finote selam district hospital
HIV: Human immunodeficiency virus
IPT: Isoniazid preventive therapy
MOH: Ministry of health
PLWHIV: People living with human immunodeficiency virus
SPSS: Statistical package for social science
TB: Tuberculosis
WHO: World Health Organization
